# Pulmonary microRNA profiles identify involvement of *Creb1* and *Sec14l3* in bronchial epithelial changes in allergic asthma

**DOI:** 10.1038/srep46026

**Published:** 2017-04-06

**Authors:** Sabine Bartel, Nikola Schulz, Francesca Alessandrini, Andrea C. Schamberger, Philipp Pagel, Fabian J. Theis, Katrin Milger, Elfriede Noessner, Stephen M. Stick, Anthony Kicic, Oliver Eickelberg, Robert J. Freishtat, Susanne Krauss-Etschmann

**Affiliations:** 1 Early origins of chronic lung disease, Priority Area Asthma & Allergy, Research Center Borstel, Leibniz-Center for Medicine and Biosciences, Airway Research Center North (ARCN), Member of the German Center for Lung Research (DZL), Borstel, Germany; 2 Comprehensive Pneumology Center (CPC-M), Institute of Lung Biology and Disease, Helmholtz Zentrum München and University Hospital of the Ludwig Maximilians University (LMU), Member of the German Center for Lung Research (DZL), Munich, Germany; 3 Children’s Hospital of the Ludwig Maximilians University, Munich, Germany; 4 Center of Allergy and Environment (ZAUM), Technische Universität and Helmholtz Zentrum München, Member of the German Center for Lung Research (DZL), Munich, Germany; 5 Numares Health, Regensburg, Germany; 6 Institute of Computational Biology, Helmholtz Zentrum München and Department of Mathematics, Technische Universität München, Munich, Germany; 7 Department of Internal Medicine V, University of Munich, Munich, Germany; 8 Institute of Molecular Immunology, Helmholtz Zentrum München, Munich, Germany; 9 Telethon Kids Institute, Centre for Health Research, The University of Western Australia, Nedlands, 6009, Western Australia, Australia; 10 School of Paediatrics and Child Health, The University of Western Australia, Nedlands, 6009, Western Australia, Australia; 11 Department of Respiratory Medicine, Princess Margaret Hospital for Children, Perth, 6001, Western Australia, Australia; 12 Centre for Cell Therapy and Regenerative Medicine, School of Medicine and Pharmacology, The University of Western Australia, Nedlands, 6009, Western Australia, Australia; 13 Division of Emergency Medicine, Children’s National Medical Center, Washington DC, USA; 14 Institute for Experimental Medicine, Christian-Albrechts-Universität zu Kiel, Kiel, Germany

## Abstract

Asthma is highly prevalent, but current therapies cannot influence the chronic course of the disease. It is thus important to understand underlying early molecular events. In this study, we aimed to use microRNAs (miRNAs) - which are critical regulators of signaling cascades - to identify so far uncharacterized asthma pathogenesis pathways. Therefore, deregulation of miRNAs was assessed in whole lungs from mice with ovalbumin (OVA)-induced allergic airway inflammation (AAI). *In silico* predicted target genes were confirmed in reporter assays and in house-dust-mite (HDM) induced AAI and primary human bronchial epithelial cells (NHBE) cultured at the air-liquid interface. We identified and validated the transcription factor cAMP-responsive element binding protein (Creb1) and its transcriptional co-activators (Crtc1-3) as targets of miR-17, miR-144, and miR-21. Sec14-like 3 (Sec14l3) - a putative target of Creb1 - was down-regulated in both asthma models and in NHBE cells upon IL13 treatment, while it’s expression correlated with ciliated cell development and decreased along with increasing goblet cell metaplasia. Finally, we propose that Creb1/Crtc1-3 and Sec14l3 could be important for early responses of the bronchial epithelium to Th2-stimuli. This study shows that miRNA profiles can be used to identify novel targets that would be overlooked in mRNA based strategies.

Asthmatic airway disease frequently begins in childhood and affects around 334 million people^1^. The resulting healthcare costs due to medication, hospitalizations, and absence from work are considerable^2^. Therapeutic options are limited and do not prevent the irreversible changes such as airway remodeling that may occur in wheezing preschool children prior to the development of asthma^3^. Thus, there is a high and unmet clinical need for new therapeutic targets and treatment strategies in asthma. A first step to address this need is to identify novel pathways that are relevant for asthma pathogenesis.

MicroRNAs (miRNAs) are a class of evolutionary highly conserved, small (20–22 nt) non-coding RNAs that post-transcriptionally control gene expression through partial binding to mRNAs. To date, 1,881 human miRNAs have been identified (www.mirbase.org), some of which are tissue or cell specific.

Differential expression of miRNAs has been observed in several complex diseases^4^ including human^5^,^6^,^7^ and experimental asthma^8^,^9^,^10^,^11^,^12^,^13^. This led to the proposal of miRNAs as therapeutic targets^8^,^9^,^11^,^14^. However, single miRNAs have numerous targets, enabling them to regulate a wide range of genes, with the potential for widespread side effects. Additionally, as the expression patterns of many miRNAs differ between disease development and disease progression^15^, targeting them in the tissue of interest and at the most appropriate time is difficult^16^.

However, single miRNAs can address several key regulatory molecules within a given cellular pathway^17^. This regulatory fine tuning at given molecular sites might result in pronounced and more focused downstream effects^18^,^19^,^20^. Therefore, we asked whether miRNAs, rather than being therapeutic targets, can facilitate the detection and prioritization of mRNA targets and pathways which might be overlooked by primarily mRNA-based or hypothesis driven identification strategies.

## Results

### Pulmonary miRNA profiling in murine allergic airway inflammation

**Figure 1:**
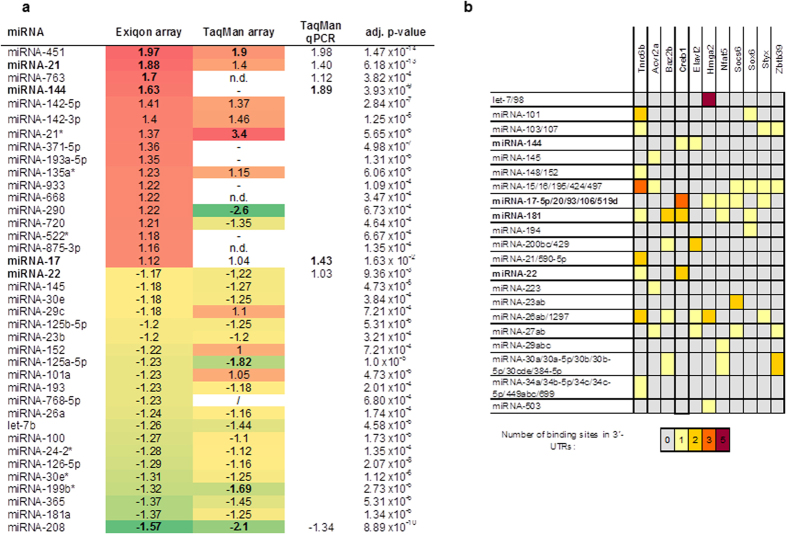
Pulmonary miRNA profile and selection of Creb1 as target gene. (**a**) Fold changes of miRNA expression analyzed by Exiqon microarray, TaqMan^®^ microarray and qRT-PCR of lung homogenate of mice with OVA-induced allergic airway inflammation and healthy controls on d72 of the treatment protocol (n = 5 mice per group) with adjusted p-value. n.d.: not detected; - miRNA not available on the array; *minor strand of miRNA. (**b)** List of genes and miRNA-binding sites in their 3′-UTRs as predicted by five different algorithms (miRanda, PicTar, PITA, TargetSpy & TargetScanS). Darker color depicts more binding sites for the same miRNA.

Female 6–8 week old Balb/c mice were intraperitoneally (i.p.) sensitized five times with ovalbumin (OVA) (d7, d14, d28, d42 and d56) (Sigma Aldrich, St. Louis, USA) or phosphate buffered saline (PBS) and challenged two times (d70 and d71) with 1% OVA aerosol to induce allergic airway inflammation. Changes in pulmonary miRNA expression were analyzed by comparing OVA/OVA exposed mice with control mice (PBS/OVA) via locked nucleic acid (LNA^TM^) microarray (Exiqon, Vedbaek, Germany). Of the 580 analyzed miRNAs, 55% were expressed in lung tissue and 37 showed a statistically significant (cut-off p-value < 10^−4^) altered expression in OVA-treated animals (Fig. 1a). The high number of “primary hits” was validated and confirmed by a PCR-based, quantitative low density array (LDA) measuring 518 murine miRNAs (TaqMan, Life Technologies, Carlsbad, USA) (Fig. 1a). From the 37 significantly regulated miRNAs (LNA^TM^ microarray) six miRNAs were not present on the LDA array, thus could not be measured, and three miRNAs were below the detection limit of the LDA array. Five miRNAs (miR-290, miR-720, miR-29c, miR-152 and miR-101a) showed inverse expressions, whereas 23 candidates showed comparable results (r = 0.62, p ≤ 0.0005; Spearman rho). Quantitative real time PCRs (qRT-PCR) of the six most significantly (p ≤ 3.9 × 10^–9^ – 1.47 × 10^−14^) dysregulated miRNAs with fold-changes >1.5 further corroborated the results of both arrays (Fig. 1a).

### Prioritization of targets addressed by differentially expressed miRNAs

We hypothesized that a number of different miRNAs, addressing several key molecules in pathways, is driving asthma pathogenesis, whereas the magnitude of single miRNA changes per se is less important. Therefore, we included the top 100 dysregulated miRNAs from the initial LNA microarray for a stringent *in silico* target prediction. Five different algorithms identified a cut-set of 961 potential target genes. We then focused on 11 genes harboring ≥4 miRNA binding sites (Fig. 1b), since binding of multiple miRNAs to the same target gene can lead to co-regulation and stronger effects on target gene expression irrespective of the degree of miRNA changes^20^. The 3′UTR of high mobility group AT-hook 2 (HMGA2) and trinucleotide repeat containing 6B (TNRC6B) showed the highest number of binding sites for single miRNAs (Fig. 1b). However, TNRC6B is an argonaute-associated protein, involved in miRNA function itself 21 and was therefore excluded. The interaction between miRNA let-7 and HMGA2 has been shown in the context of lung cancer^22^ and let-7 has been intensively studied in AAI already^13^. Based on the following considerations, we chose the transcription factor *Creb1* for further investigation: First, we assumed strong control as judged by the high number of miRNA-binding sites (Fig. 1b); second, Creb1 is important for the regulation of immune responses^23^; and third, it is decreased in response to Th2-stimuli^24^. In total, the 3′UTR of *Creb1* contains eight predicted binding sites for miR-17 (three sites), miR-144 (one site), miR-22 (two sites), and miR-181a (two sites) (Fig. 1b). Furthermore, previous reports suggested a role for Creb1 in asthma and COPD^25^,^26^,^27^.

### *In vitro* validation of predicted miRNA targets

**Figure 2:**
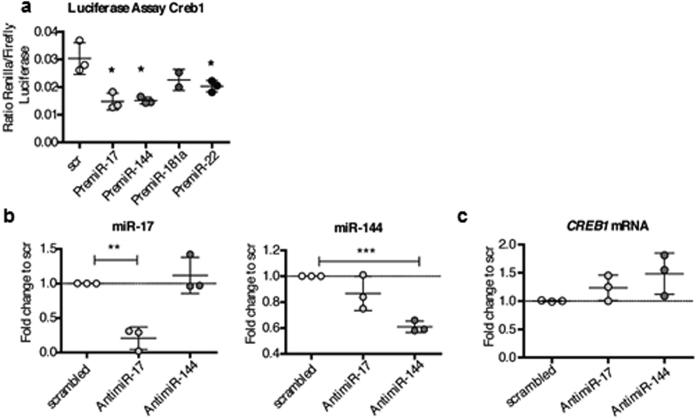
miRNA-regulation of CREB1 *in vitro.* Transfection of 16-HBE14o^−^ cells with **(a)** PremiRs or scrambled miRNA and a luciferase-vector containing the Creb1 3′-UTR fused to Renilla luciferase, representative of two independent experiments with n = 3 wells. **(b,c)** AntimiR transfection (n = 3). **(b)** qRT-PCR for miRNAs and **(c)** Creb1. All mean ± SD, *p < 0.05; **p < 0.01, ***p < 0.001 vs. scrambled control.

The functional interaction between *Creb1* and miR-17, -144, and -22 was confirmed by luciferase reporter assays (Fig. 2a). Since binding of miR-181a could not be proven (Fig. 2a), and miR-22 and could not be confirmed by qRT-PCR, we excluded both miRNAs from further analysis. Antagonism of miR-17 or -144 *in vitro* via transfection of anti-miRs slightly increased CREB1 mRNA levels (Fig. 2b).

After phosphorylation, Creb1 has been shown to bind to cAMP-responsive element (CRE) sites in the promoter region of target genes^28^. Transcriptional activity of Creb1 is enhanced by binding of its co-activators, the Creb-regulated transcriptional co-activators (Crtcs)^29^. We therefore evaluated whether joint down-regulation of Crtc proteins and Creb1 by the candidate miRNAs has an additive effect on the transcription of Creb1 downstream targets. A less stringent i*n silico* analysis with only two different target prediction tools identified miR-144 and miR-17 (*homo sapiens* only) binding sites in the 3′-UTRs of *Crtc1* and *Crtc3* (Table S1). miRNA-21 was included *post hoc* as it was not only predicted to target *Crtc1* and *Creb1*, but was also the second highest “hit” in our array (adj. p-value 6.18 × 10^−13^). Further, other researchers have suggested that it has a role in allergic airway inflammation^11^,^30^.

*In vitro, Crtc2* levels were significantly reduced by transfection of a miRNA mimic (premiR) of all three microRNAs. *Crtc3* was reduced by miR-144 and miR-21 over-expression, whereas *Crtc1* levels where only reduced by miR-21 (Fig. S1a). miR-17 also decreased *Crtc2* (Fig. S1a). Antagonism of endogenous miR-17 and -144 (Fig. S1b) slightly increased *CRTC1*, -2, and -3 mRNA levels (Fig. S1b) levels.

### Inverse regulation of miRNA and Creb1/Crtc1-3 in allergic airway inflammation

**Figure 3:**
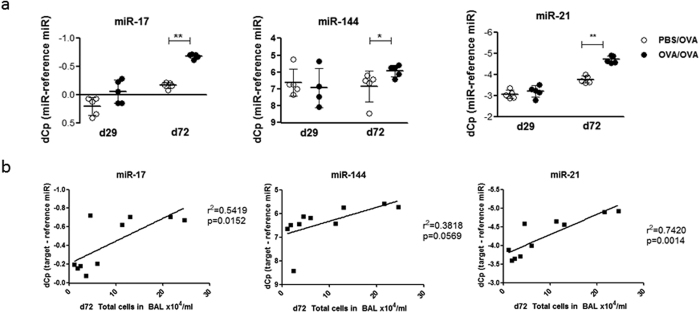
miRNA regulation in murine allergic airway inflammation. (**a**) Expression analysis via qRT-PCR for miRNAs of lung homogenates of PBS/OVA and OVA/OVA mice (n = 5 mice per group), y-axis depicts dCp values (Cp_miRNA_-Cp_reference miR_) – higher dCp means lower expression (reversed scale), Mann-Whitney U, *p < 0.05; **p < 0.01 vs. PBS/OVA control. (**b)** Correlations of miRNA dCp values with total cell counts from bronchoalveolar lavage (BAL).

As these results suggested that miRNA-17, -144, and -21 regulate Creb1 and its co-activators, we sought to verify this regulation during the development of OVA-induced AAI. In lung homogenates of OVA/OVA treated mice, the levels of all three candidate miRNAs significantly increased after the allergen challenge (day 72), but not during sensitization (day 29) (Fig. 3a). Additionally, the levels of miR-17, miR-144 and miR-21 significantly correlated with total cell counts in bronchoalveolar lavage (BAL), indicating an increased expression of the miRNAs upon increasing inflammation (Fig. 3b).

**Figure 4:**
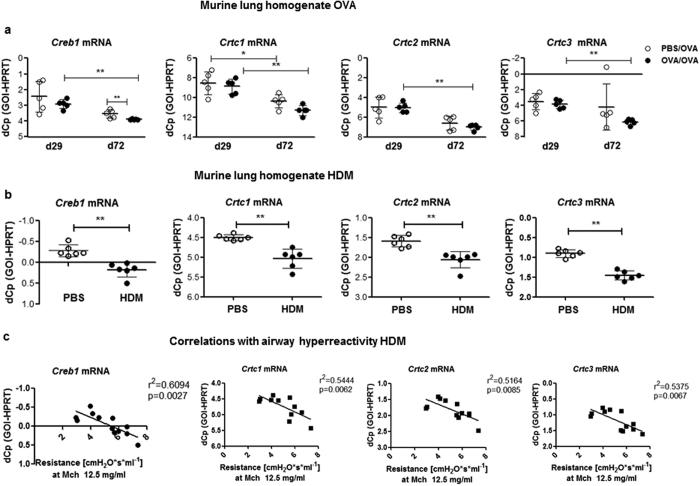
Down-regulation of Creb1/Crtc expression in murine allergic airway inflammation. Expression analysis via qRT-PCR for Creb1, Crtc1, Crtc2 and Crtc3 in **(a)** lung homogenates of PBS/OVA and OVA/OVA mice (5 mice per group), **(b)** lung homogenates of HDM-treated mice vs. PBS-treated controls (6 mice per group). (**a,b**) y-axis depicts dCp values (Cp_gene_-Cp_reference gene_) – higher dCp means lower expression (reversed scale). All mean ± SD, Mann-Whitney U, *p < 0.05; **p < 0.01 vs. respective controls. (**c**) Correlations of Creb1 & Crtc1, Crtc2 and Crtc3 dCp values with airway hyperreactivity (Ahr) as depicted by resistance to 12.5 mg/ml methacholine.

*Creb1* mRNA was decreased in lung homogenate of those OVA/OVA treated mice on day 72 compared to PBS-treated controls (Fig. 4a). During OVA challenge the transcript levels of *Creb1* and all three *Crtcs* decreased from day 29 to day 72; in control animals this was only seen for Crtc1 (Fig. 4a). The protein levels of Crtc1 and Crtc3 were assessed by western blotting and increased from day 29 to day 72 in control mice (Fig. S2a). This increase was significantly attenuated for Crtc3 following OVA challenge.

To further validate these findings in a model with more relevance for human asthma we used house dust mite (HDM) induced experimental asthma. Briefly, female 6–8 week old Balb/c were treated intranasally three times per week over five weeks with 20 μg Dermatophagoides pteronyssinus extract (Nr. 218234; Greer, Lenoir, USA). *Creb1* and *Crtc1, 2 and 3* mRNA levels were significantly decreased in HDM-treated animals (Fig. 4b), and negatively correlated with increased airway hyperreactivity (Fig. 4c). HDM-treatment also significantly reduced Crtc3, with a trend towards reduction in Creb1 protein levels (Fig. S2b).

### The *Creb1* transcriptional target *Sec14l3* is decreased in murine AAI

**Figure 5:**
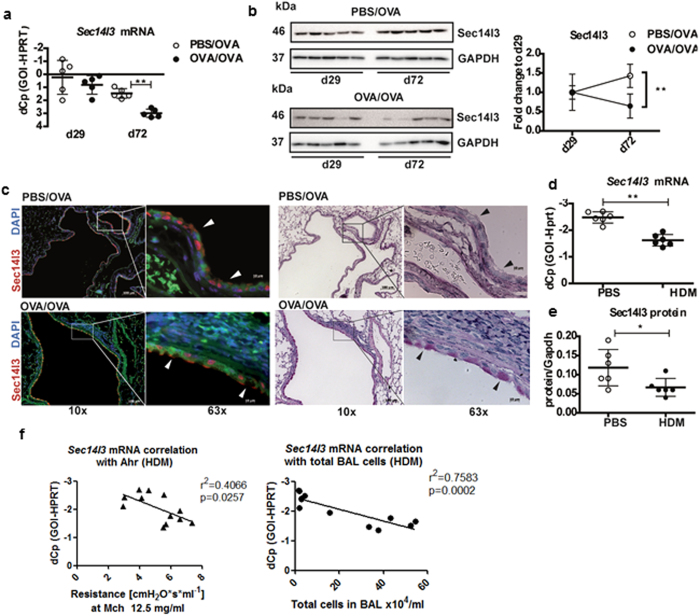
Sec14l3 in murine allergic airway inflammation. (**a**) qRT-PCR and (**b**) Western blot and densitometrical analysis of OVA-treated animals, mean ± SD, (n = 5 mice/ group). The blot was cropped to improve clarity – full-length blots are provided in Supplemental Fig. S3 (**c**) Representative lung sections: Sec14l3 (red) vs. DAPI (blue), (left) or PAS (right). Arrowheads: Sec14l3 + cells (left) and goblet cells (right) (4 mice/group). HDM-treated mice (n = 6 mice/group), mean ± SD **(d)** qRT-PCR. (**a,d**) y-axis depicts dCp values (Cp_gene_-Cp_reference gene_) – higher dCp means lower expression (reversed scale) (**e**) densitometrical analysis of Western blot. *p < 0.05; **p < 0.01 vs. PBS. (**f**) Correlations of Sec14l3 mRNA (dCp values) with total cell counts in BAL and airway hyperreactivity (Ahr) as depicted by resistance to 12.5 mg/ml methacholine.

As Creb1 and the Crtcs were altered in the both murine AAI models, we asked whether their down-regulation also negatively influences the transcription of Creb1 target genes. To this end, we searched an mRNA array previously performed (using lung homogenate collected on d72) of our OVA model^31^ (GEO database, ID: GSE6496 (http://www.ncbi.nlm.nih.gov/geo) for down-regulated genes containing CRE-sites in OVA/OVA vs. PBS/OVA mice. Of 185 decreased genes, 35 contained putative CRE-elements (Table S2). We selected Sec14-like 3 (Sec14l3) for further investigation as it had already been found to be decreased in allergic airway inflammation in rats^32^ and to be specifically expressed in murine airway ciliated cells^33^. *Sec14l3* mRNA levels were decreased in lungs of OVA-animals (Fig. 5a). Similar to Creb1 and the Crtcs, Sec14l3 protein levels increased in PBS/OVA mice, but the increase was abolished in animals with OVA-induced allergic inflammation (Fig. 5b). Immunofluorescence staining demonstrated Sec14l3 expression in airway ciliated cells of healthy animals, with lower levels in OVA-induced AAI (Fig. 5c). PAS staining of matching lung sections showed a concomitant goblet cell metaplasia (Fig. 5c). These findings were corroborated in HDM-induced AAI (Figs 5d,e and S4). In the HDM model, *Sec14l3* mRNA levels also significantly and negatively correlated with increasing airway hyperreactivity and total cell counts in the BAL (Fig. 5f). This suggested that loss of Sec14l3 is associated with AAI, independent of the type of allergen.

### IL13 induced goblet cell metaplasia in primary NHBE cells decreases *SEC14L3* expression

**Figure 6:**
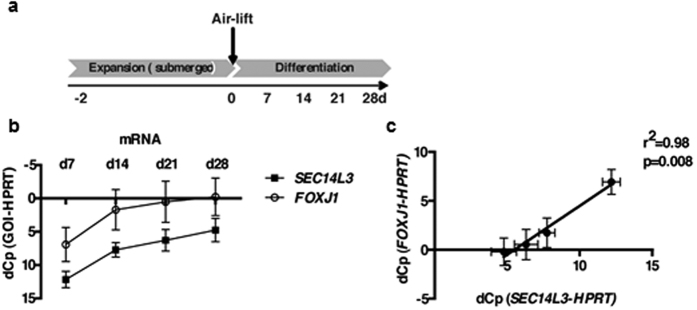
*SEC14L3* increases during NHBE differentiation and correlates with *FoxJ1*. (**a**) Treatment scheme of differentiation of primary normal human bronchial epithelial cells (NHBE) into a pseudostratified epithelium at the air-liquid interface. **(b)** qRT-PCR for SEC14L3 and FOXJ1 from day 7 to day28 of differentiation (n = 4 independent experiments). y-axis depicts dCp values (Cp_gene_-Cp_reference gene_) – higher dCp means lower expression (reversed scale). **(c)** Linear correlation of dCp values of SEC14L3 and FOXJ1 (n = 4 independent experiments).

In order to get further insight into the regulation of epithelial *SEC14L3* in the context of allergic airway inflammation, we differentiated primary normal human bronchial epithelial (NHBE) cells (Lonza, Basel, Switzerland) at the air-liquid interface in PneumaCult™-ALI medium (Stemcell Technologies; Köln, Germany) containing 1% penicillin/streptomycin at 5% CO_2_ and 37 °C. After 28 days, a pseudostratified epithelium was formed, containing goblet, club, ciliated and basal cells^34^. To induce early asthma-like changes in the bronchial epithelium, the cells were treated with 10 ng/mL of the potent Th2 cytokine IL13 (R&D Systems, Wiesbaden, Germany) from the basolateral side between d0 and d7 (Fig. 6a).

On day 0, *SEC14L3* mRNA was undetectable, but levels increased markedly until day 28 (Fig. 6b). Of note, this increase closely correlated with mRNA expression of the transcription factor *Forkhead box protein J1 (FOXJ1*) which is associated with the development and function of ciliated cells^35^ (Fig. 6c).

**Figure 7:**
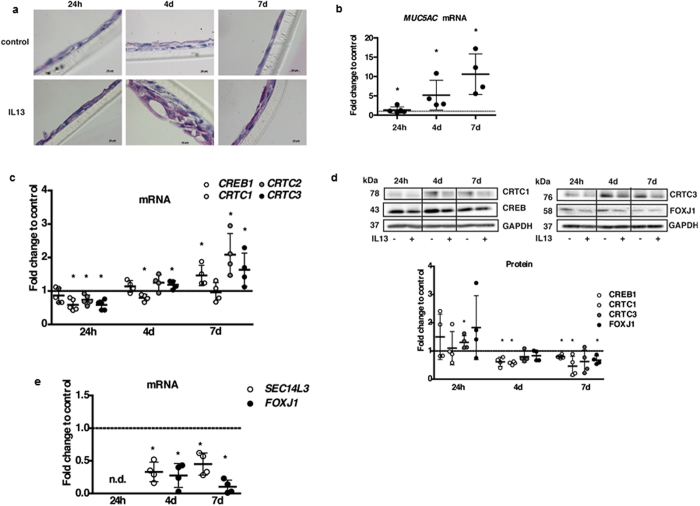
IL13 treatment of NHBE cells reduces *CREB1* and *SEC14l3*. (**a**) PAS staining of transwell membranes. **(b)** qRT-PCR for MUC5AC. **(c)** qRT-PCR for CREB1 and CRTC1-3. (**d**) Representative western blot and densitometrical analysis. The blot was cropped to improve clarity – full-length blots are provided in Supplemental Fig. S5 (**e**) qRT-PCR for SEC14L3 and FOXJ1. All graphs depict fold change vs. control. n.d. = not detectable. All n = 4, Mann-Whitney U, *p < 0.05 vs. untreated control.

Induction of goblet cell metaplasia was confirmed by a cross-section PAS staining of the transwell membranes (Fig. 7a) and *Mucin 5A/C (MUC5AC)* mRNA increase (Fig. 7b). The mRNA of *CREB1 and its* co-activator *CRTCs* decreased significantly after only 24 hours of IL13 treatment, but then increased over time (Fig. 7c). These findings were largely translated to the protein level (Fig. 7d), although with more prolonged decreases. *SEC14L3* and *FOXJ1* transcript levels were significantly decreased upon IL13 treatment (with levels not detectable at 24 h), abolishing the increase observed during normal differentiation (Fig. 7e).

Overall, changes in the bronchial epithelium in response to Th2 stimuli seem to disturb *CREB1*/*CRTC*-mediated transcription of its putative target *SEC14L3*.

## Discussion

Several studies have shown that modulation of single miRNAs such as miR-21^11^,^36^, let-7d^13^ and miR-145^8^ inhibits or ameliorates the development of allergic airway inflammation in mice. In this study, rather than studying single miRNAs during asthma development, we used profiles of dysregulated miRNA as a tool to identify novel, regulatory gene networks of relevance for the pathogenesis of asthma that might be overlooked by mRNA array based studies. We identified down-regulation of Creb1/Crtc-mediated transcription and its putative target Sec14l3 in two murine experimental models, independent from the type of allergen. In primary NHBE cells, we demonstrated that soon after IL13 exposure *CREB1* and *CRTC* levels were decreased. Th2 stimulus also decreased *SEC14L3* and *FOXJ1* expression, which was associated with goblet cell metaplasia.

Altered expression of miRNAs have been observed in bronchial biopsies of patients with asthma^6^,^7^. Previous researchers have proposed the miR-34/449 family^6^ and miR-146a^7^ to regulate epithelial changes in human asthma, influenced by cytokine stimulation in human bronchial epithelial cells. However, neither miR-146a nor the 34/449 family were significantly altered in our array. This might be explained by the fact that the prior studies investigated miRNAs in isolated human bronchial epithelial cells, whereas we conducted the miRNA profile analysis in murine lung homogenate.

Up-regulation of miR-21 with repression of *IL-12p35* has previously been demonstrated in three different animal models of allergic airway inflammation^11^, and recently also in severe steroid-insensitive asthma^36^. The miR17-92 family is broadly expressed from earliest development to adulthood and plays a central role in lung development^37^. So far, dysregulation of miR-17 has been mainly investigated in the context of cancer^38^,^39^, but has also been proposed to regulate IL-10 in regulatory T cells^40^. The transcription of IL10 has also been shown to be regulated by Creb and Crtc3 in regulatory macrophages^41^. Thus, the miRNA-mediated down-regulation of Creb1 and Crtc3 in AAI, combined with the up-regulation of miR-17, could influence the secretion of the anti-inflammatory cytokine IL10 and could therefore have a beneficial impact on airway inflammation. Recently, miR-17/20 has also been shown to regulate pulmonary artery smooth muscle cell proliferation via targeting prolyl hydroxylase 2, leading to induction of hypoxia-inducible factor (HIF) 1α^42^. Of note, HIF1α deletion in mice has been shown to ameliorate OVA-induced asthma with lower eosinophil infiltrations, reduced goblet cell hyperplasia and lower Th2 cytokine levels. Thus, it is intriguing to speculate that increased miR-17 levels in our model might induce HIF1α levels, contributing to allergic airway inflammation.

To our knowledge the involvement of miR-144 has not been proposed in allergic airway inflammation so far. This miRNA is abundantly expressed in red blood cells^43^, but has recently been investigated in lung cancer biology^44^,^45^, showing a functional expression in lung cells.

It should be noted that in our series of experiments we only sought to use miRNA profiles to identify dysregulated asthma profiles. Further work would be needed to provide functional proof that these pathways are involved in asthma pathogenesis, and indeed this would be a most intriguing area for future investigation.

In our acute murine models *Creb1* and *Crtc* down-regulation was detected directly after allergen challenge and further, human *CREB1*/*CRTCs* were significantly down-regulated in primary NHBE cells early after IL13 treatment, but returned to normal after 7 days. Therefore, we speculate that an initial but transient dysregulation of CREB1 and CRTC-mediated transcription is sufficient to induce lasting changes of downstream targets in asthma (either directly or indirectly).

Our findings run partly counter to two reports, one showing an increase of pulmonary phosphorylated (p)CREB in adult steroid-resistant or untreated asthma^25^, and the other reporting an increase of total CREB in peripheral blood of patients with recurrent wheeze^46^. However, in a novel analysis of published gene expression data (DEGAS = DysrEgulated Gene set Analysis via Subnetworks) with the aim to identify subnetworks and dysregulated pathways in diseases, CREB1 was listed as down-regulated in airway epithelial cells of patients with asthma, confirming our finding from primary NHBE cells^47^,^48^. Further, CREB1 binding activity negatively correlated with recurrent airway obstruction in horses, which have many similarities with human asthma^26^. Similar to our findings with IL13, the Th2-associated chemokine CCL17 has been shown to down-regulate CREB1 *in vitro*^24^. Another study has proposed CREB1 to be influenced by IL17, a cytokine that is implicated in the pathogenesis of severe asthma^49^. Thus, the role of CREB1 in asthma is not yet clear and requires further investigation.

Crtc proteins have so far been mainly described in the context of regulation of metabolism. With the exception of one study that suggested Crtc3 promotes IL10 synthesis^41^, reports on Crtcs in immune regulation are scarce^50^. The involvement of Crtcs in asthma or any other lung disease has to our knowledge not been investigated so far.

The lipid transporter Sec14l3 has been proposed as being important for the maintenance and homeostasis of rat airway epithelial cells^32^. In our study, *Sec14l3* was strongly down-regulated in ciliated cells of animals with experimental AAI. We do not have direct proof that Creb1 binds to the promoter of Sec14l3 as the latter gene is not expressed in lung cell lines, impeding siRNA studies. Nonetheless, the CRE-element in the Sec14l3 promoter is evolutionary highly conserved, suggesting functional relevance. Little is known regarding functions of Sec14l3, which is highly induced around birth in lung tissue^51^. In mammalians more than 20 Sec14 family members have been described, carrying highly conserved lipid-binding domains^52^. Sec14l3 has been specifically implicated in the intracellular transport of α-tocopherol^53^ and has been proposed as sensor of liposomal lipid-packing defects in the lung^51^. Human SEC14L3 has been found to be decreased in bronchial and nasal epithelium of smokers^54^ and during human rhinovirus infection^55^. In the present work, loss of SEC14L3 expression in ciliated cells in mouse lungs and human primary bronchial cell cultures correlated with goblet cell metaplasia. Thus, the SEC14L3 expression profile could mirror cellular changes in the epithelium.

In summary, we have identified a dysregulation of Creb1, the Crtcs (1–3) and their transcriptional target Sec14l3 in early stages of asthma pathogenesis. Thus, we suggest that miRNA profiles can be used as tool for prioritization of disease-relevant pathways.

## Materials and Methods

### Animals

Female Balb/c mice were obtained from Charles River (Sulzfeld, Germany) and housed in individually ventilated cages. A standard pellet diet and water were provided *ad libitum*. The study was conducted under the German federal guidelines for the use and care of laboratory animals and was approved by the Government of the District of Upper Bavaria.

### Ovalbumin-induced allergic airway inflammation

Female 6 to 8 weeks old Balb/c mice were intraperitoneally (i.p.) sensitized with 1 μg ovalbumin (OVA) (Sigma Aldrich, St. Louis, USA) (or phosphate buffered saline (PBS) for controls) in alum, followed by two aerosol challenges with 1% OVA for 20 min (Fig. S6a) as previously described^56^. Mice were sacrificed on day 29 or 72 (Fig. S6a). The ability of the model to generate moderate allergic airway inflammation was verified by high levels of OVA-specific serum IgE and IgG1 (Fig. S6b), elevated numbers of inflammatory cells in bronchoalveolar lavage fluid (BALF) (Fig. S6c,d), elevated titers of CCL17 in BALF (Fig. S6e), and goblet cell metaplasia in the lungs (Fig. S6f).

### RNA extraction and quality analysis

Total RNA (including small RNAs) was isolated from homogenized lung tissue or cell culture using the miRNeasy Mini Kit according to the manufacturer’s instructions (Qiagen, Venlo, The Netherlands). Concentrations were determined using a NanoDrop^®^ ND-1000 spectrophotometer (NanoDrop Technologies, Erlangen, Germany). Quality was assessed by gel electrophoresis and by using the Agilent 2100 bioanalyzer (Agilent Technologies, Santa Clara, USA).

### Locked nucleic acid miRNA microarray

Hy3-/Hy-5 labelled RNA (miRCURY LNA microRNA Array Power Labeling Kit, Exiqon, Vedbaek, Germany) was manually hybridized on miRNA microarrays (miRCURY^TM^ LNA microRNA Array v10, Exiqon), which comprised 580 murine miRNAs (miRBase release 10.0). Slides were scanned by a GenePix 4000A Microarray Scanner (Axon Instruments, Foster City, USA) and analyzed by GenePix^®^ Pro Software (Axon Instruments). Differential expression of miRNAs was identified using a linear model approach (limma, Bioconductor analysis suite^57^). After background subtraction, non-positive spots were removed and the remaining signal intensity values were normalized to a small control RNA (RNU6B) (Life technologies, Carlsbad, USA). P-values were adjusted to multiple testing by Bonferroni correction.

### Low density array

For validation of the microarray data, TaqMan^®^ Array Rodent MicroRNA Cards (Applied Biosystems, Life Technologies, Carlsbad, USA) were performed according to the manufacturer’s protocol with an input of 350 ng total RNA per array card and pre-amplification procedure. In total 518 murine miRNAs were measured. The array version 2.0 (miRBase v.10.0) was run on an ABI PRISM^®^ 7900HT real-time PCR machine (Applied Biosystems, Carlsbad, USA) and analyzed by SDS-software (SDS v2.2) via a comparative cycle threshold method (∆∆Ct)^58^.

### Microarray statistics

Statistical analysis was carried out with the data analysis and statistics language R (R Foundation for Statistical Computing) using the Bioconductor suite for bioinformatics^59^, specifically the limma package^57^,^60^ which fits a linear model for each gene and computes a moderated *t*-statistic and its *P* value^61^. Background correction was done using the normexp method by Ritchie *et al*.^62^. Within-array normalization was carried out by robust spline normalization of the log R/G ratio and scaling was used for between-array normalization. *P* values were corrected for multiple testing by the method of Benjamini and Hochberg^63^.

### Quantitative real-time PCR (qRT-PCR)

Expression of single miRNAs was evaluated using TaqMan-based specific real-time PCR (Applied Biosystems, Carlsbad, CA, USA) following the manufacturer’s instructions and normalized to small control RNA sno-234 (Life technologies) for murine tissue and murine cell lines and RNU-6B (Life technologies)for human cells lines and nasal brushings. mRNA was converted to cDNA with the QuantiTect Rev. Transcription Kit (Qiagen, Venlo, The Netherlands). PCRs were performed on the LightCycler 480 platform (Roche, Mannheim, Germany) using LightCycler 480 SYBR Green I Mastermix or for miRNAs TaqMan^®^ Universal PCR Master Mix (Invitrogen, Carlsbad, USA). Expression differences were calculated based on the ∆∆Ct method using HPRT as reference gene^58^. Specific primer sequences for *CREB1, CRTC1*-*3, SEC14L3, FOXJ1* and *MUC5AC* are provided in the Table S3.

### *In silico* target predictions

A strict “full consensus” approach using five different prediction algorithms was applied: miRanda: (http://www.microrna.org/microrna/getDownloads.do); PicTar (www.pictar.org); PITA: (genie.weizmann.ac.il/pubs/mir07/mir07_prediction.html), Targetspy (http://webclu.bio.wzw.tum.de/targetspy/index.php?down=true); Target-ScanS: (genes.mit.edu/targetscan). Only fully-consensus predicted targets were considered for initial target prioritization. We therefore included a second step in which we critically reviewed miRNAs that had been excluded from the full-consensus algorithm approach, but that targeted Crtc1, Crtc2 or Crtc3. Thus, miRNAs that only met two of the algorithms (miRanda and PITA), and that are known to be biologically relevant and highly deregulated, were also included, providing their binding was subsequently verified *in vitro*. For a complete list of predicted miRNA binding sites in the Crtc1, Crtc2 and Crtc3 genes see Table S1”.

### Cell culture

Murine lung epithelial cells (MLE-12) and human bronchial epithelial cells (16-HBE14o^−^)^64^ were cultured in MEM medium supplemented with 10% FCS at 37 °C and 5% CO_2_ without antibiotics under standard conditions.

### Luciferase reporter assay

The complete *Creb1* 3′untranslated region (UTR) was amplified from mouse genomic DNA (see Table S3 for primer sequences) and cloned into psiCHECK-2 vector (Promega, Madison, USA). Fifty ng of the vector construct were transfected into 16-HBE14o^−^ cells together with the precursor miRNAs of interest or a negative control miRNA. After 72 h, cells were lysed (200 mM Tris-HCl, 0.1% Triton in 500 ml H_2_O, pH 7.4) and *Renilla* and *firefly* luciferase activities were measured (Wallac 1420 Multilabel counter, Perkin Elmer, Waltham, USA) after addition of the respective substrates. *Renilla* luciferase activity was normalized to firefly luciferase activity.

### Transfection assays

Transfection experiments were conducted with Lipofectamine 2000 (Invitrogen, Carlsbad, USA) in 12-well plates following manufacturer’s instructions. Ambion^®^ Pre-miR Precursors (for miR-17 and miR-144), miRvana miRNA mimics (for miR-21) (Ambion, Austin, USA) or antimiRs (miR-17 and -144) (Ambion, Austin, USA) were transfected in duplicates to a final miRNA concentration of 20 nM per well in a murine lung epithelial cell line (MLE-12) or a human bronchial epithelial cell line (16-HBE14o^−^)^64^. Analyses were done 72 h after transfection for Pre-miRNA or 24 h for antimiR transfection.

### Western blotting and densitometry

Lung tissue or cells from culture were homogenized in RIPA buffer containing 10 mM NaF, 1 mM Na_3_VO_4_, 1 mM DTT and protease inhibitor and sonicated for 10 sec to disrupt cellular structures. Protein concentrations were measured using Pierce BCA protein assay kit (Thermo Fischer Scientific, Waltham, USA) following the manufacturer’s protocol. 20 μg protein per well were separated by SDS-Polyacrylamid-gel-electrophoresis and transferred to PVDF membranes by wet blotting (BioRad, Hercules, USA). Membranes were incubated under constant agitation over night at 4 °C with antibodies against Creb1, Crtc1, Crtc3, Sec14l3 (Abcam, Cambridge, USA) or Gapdh antibody as loading control (all Cell signalling Technology, Danvers, USA) (all 1:1000 in 5% BSA in TBS-T) and for 1.5 h at room temperature with secondary HRP-conjugated goat anti-rabbit IgG antibody (BioRad, Hercules, USA) (1:40000, 5% BSA in TBS-T). ECL, Dura-ECL and Femto-ECL (Thermo Fischer Scientific, Waltham, USA) were used for detection via Chemidoc XRS+ Molecular Imager (BioRad, Hercules, USA). For reprobing, membranes were stripped in Restore Plus Stripping buffer (Thermo Fischer Scientific, Waltham, USA) according to the manufacturer’s recommendations. Quantification of band intensities was performed with the Image Lab software v4.01 (BioRad, Hercules, USA).

### House dust mite induced allergic airway inflammation

For house dust mite (HDM) induced AAI, mice were treated intranasally 3 times per week over 5 weeks with 20 μg Dermatophagoides pteronyssinus extract (Nr. 218234; Greer, Lenoir, USA) in 30 μL PBS or PBS (Fig. S7a). HDM-induced allergic airway inflammation was validated by BALF cell counts (Fig. S7b), differential cell counts (Fig. S7c), increased airway hyper-reactivity in response to metacholine (Fig. S7d), and lung inflammatory cell infiltration (Fig. S7e).

### Lung function

Animals were i.p. anesthetized with ketamine (140 mg/kg) and xylazine (7 mg/kg), tracheostomized, intubated (18G tube), placed on a warming plate and ventilated with a tidal volume of 10 mL/kg at a frequency of 150 breaths/minute and a positive end-expiratory pressure of 2 cm H_2_O on a Buxco R/C system. To assess airway hyperreactivity, the mice were challenged with metacholine in physiological saline generated with an in-line nebulizer and administered directly with increasing concentrations (0, 6.25, 12.5 mg/mL) for 20 seconds. The highest values of respiratory system resistance were recorded continuously for 2 min after each metacholine dose, the average was calculated and plotted against metacholine concentration.

### *Creb1* target gene search

The palindromic cAMP responsive elements (CRE)-elements in the promoter region of potential Creb1 targets (5′-TGACGTCA-3′) were identified by using “MatInspector Release professional 8.0.5, March 2011 (Database version: ElDorado 08–2011; MatInspector library: Matrix Family Library Version 8.4” (June 2011), Table S2).

### Lung histology and Immunofluorescence staining

Goblet cell hyperplasia was assessed by a periodic acid Schiff stain (PAS) in formalin-fixed, paraffin-embedded lung tissue sections. Sec14l3 expression was determined by immunofluorescence using a specific rabbit anti-rat Sec14l3 antibody (Abcam, Cambridge, MA, USA) (1:200) and Alexa Fluor 568 goat anti-rabbit IgG (H+L) (Life technologies, Carlsbad, USA) (1:250). Nuclei were co-stained with DAPI (1:2500, 0.5 μg/mL). Specific binding of murine Sec14l3 was verified by western blot beforehand.

### Primary normal human bronchial epithelial cell differentiation and IL13 treatment

Primary human bronchial epithelial (NHBE) cells from different healthy donors (Lonza; Wokingham, UK) were cultivated in bronchial epithelial cell growth medium (BEGM) (Lonza; Wokingham, UK) as described previously^34^. NHBE cells were differentiated up to 28 days at the air-liquid interface (ALI) using PneumaCult™-ALI medium (Stemcell Technologies; Köln, Germany) containing 1% penicillin/streptomycin. For induction of goblet cell metaplasia, NHBE cells were exposed to 10 ng/mL IL13 (R&D systems, Wiesbaden, Germany) between day 0 and day 7 of ALI-culture from the basolateral side of the transwell. IL13 treatment was renewed every 2–3 days with simultaneous changes of the growth medium. For cross-section histology, the membranes were fixed with 4% paraformaldehyde (PFA) and embedded in 2% agarose in Hank’s balanced salt solution (HBSS) prior to paraffin embedding.

### Statistics

Unless stated otherwise, the non-parametric Mann-Whitney *U* test was applied for animal experiments and cell cultures with n ≥ 4, and ANOVA plus Bonferroni for lung function measurements with *p ≤ 0.05, **p ≤ 0.01; ***p ≤ 0.001. Data are presented as mean ± SD (GraphPad Prism 5 software; La Jolla, CA, USA).

## Additional Information

**How to cite this article:** Bartel, S. *et al*. Pulmonary microRNA profiles identify involvement of *Creb1* and *Sec14l3* in bronchial epithelial changes in allergic asthma. *Sci. Rep.* **7**, 46026; doi: 10.1038/srep46026 (2017).

**Publisher's note:** Springer Nature remains neutral with regard to jurisdictional claims in published maps and institutional affiliations.

## Supplementary Material

Supplementary Information
