# Supplementary material for: Pulmonary microRNA profiles identify involvement of Creb1 and Sec14l3 in bronchial epithelial changes in allergic asthma
Source: Sci Rep. 2017 Apr 6;7:46026. doi: 10.1038/srep46026 (PMC5382551; doi:10.1038/srep46026)
Supplement: Supplementary Information [file srep46026-s1.pdf]

SUPPLEMENTARY INFORMATION

Pulmonary microRNA profiles identify involvement of *Creb1* and *Sec14l3* in bronchial epithelial changes in allergic asthma

Sabine Bartel[1,2*] and Nikola Schulz[2-4*], Francesca Alessandrini[5#] and Andrea C. Schamberger[2#], Philipp Pagel[6], Fabian J. Theis[7], Katrin Milger[2,3,8], Elfriede Noessner[9], Stephen M. Stick[10,11,12,13], Anthony Kicic[10,11,12,13], Oliver Eickelberg[2,3], Robert J. Freishtat[14], Susanne Krauss-Etschmann[1-4,15]

*, # Equal contribution

[1]Experimental Asthma Research, Priority Area Asthma & Allergy, Research Center Borstel, Leibniz-Center for Medicine and Biosciences, Airway Research Center North (ARCN), Member of the German Center for Lung Research (DZL), Borstel, Germany

[2]Comprehensive Pneumology Center (CPC-M), Institute of Lung Biology and Disease, Helmholtz Zentrum München and [3]University Hospital of the Ludwig Maximilians University (LMU), Member of the German Center for Lung Research (DZL), Munich, Germany

[4]Children's Hospital of the Ludwig Maximilians University, Munich, Germany.

[5]Center of Allergy and Environment (ZAUM), Technische Universität and Helmholtz Zentrum München, Munich, Germany

[6]Numares Health, Regensburg, Germany

[7]MIPS, Institute for Bioinformatics and System Biology, Helmholtz Zentrum München, Neuherberg, Germany

[8]Department of Internal Medicine V, University of Munich

[9]Institute of Molecular Immunology, Helmholtz Zentrum München, Munich, Germany

[10]Department of Respiratory Medicine, Princess Margaret Hospital for Children, Perth, 6001,Western Australia, Australia. [11]School of Paediatrics and Child Health! The University of Western Australia, Nedlands, 6009, Western Australia, Australia.[12]Telethon Kids Institute, Centre for Child Health, the University of Western Australia, Nedlands, 6009, Western Australia, Australia. [13]Centre for Cell Therapy and Regenerative Medicine, School of Medicine and Pharmacology, the University of Western Australia, Nedlands, 6009, Western Australia, Australia.

[14]Division of Emergency Medicine, Children's National Medical Center, Washington DC, USA

[15]Institute for Experimental Medicine, Christian-Albrechts-Universität zu Kiel, Kiel, Germany

RESULTS

SUPPLEMENTARY TABLES

miRNA	CRTC1 (hsa)	CRTC2 (hsa)	CRTC3 (hsa)	SEC14L3 (hsa)
miR-17	- (+)	- (-)	- (+)	- (-)
miR-144	+ (+)	+ (-)	+ (+)	- (+)
miR-21	+ (+)	- (-)	- (+)	- (-)

Table S1. Potential miRNA binding sites within the 3’UTR of candidate genes predicted by miRanda and PITA target prediction tools. (-) Absence or (+) presence of miRNA binding site in 3’UTR

Gene symbol	GeneID	logFC (OVA/OVA vs PBS/OVA)	Gene symbol	GeneID	logFC (OVA/OVA vs PBS/OVA)
Dcpp1	13184	-2.688	Mapk3	26417	-1.071
Tnnt3	21957	-2.296	Ltbp4	108075	-1.053
Tnni2	21953	-1.889	Ppp1cb	19046	-1.051
Ddx5	13207	-1.842	Rsrc2	208606	-1.043
Tnnc2	21925	-1.801	Tap1	21354	-1.018
Car3	12350	-1.765	Akap3	11642	-1.015
Lor	16939	-1.711	Tcp1	21454	-1.006
Ghitm	66092	-1.692	Krt14	16664	-1.004
Calml3	70405	-1.622	Nup155	170762	-0.954
Sec14l3	380683	-1.341	Dnajb6	23950	-0.942
Gpd1	14555	-1.292	Mark3	17169	-0.913
Tubb2c	227613	-1.209	Cask	12361	-0.887
Rbbp7	245688	-1.204	Lypd3	72434	-0.886
Caprin1	53872	-1.170	Cidea	12683	-0.857
Son	20658	-1.165	Sypl	19027	-0.855
Ddx17	67040	-1.149	Atp8a1	11980	-0.836
Cap2	67252	-1.127	Fabp5	16592	-0.833
Mgll	23945	-1.092			

Table S2. Dysregulated putative CREB target genes in OVA-induced asthma. Degree of regulation is listed as log fold change vs. PBS/OVA.

Gene name	Species	fwd 5'->3'	rev 5'->3'
CRTC1	hsa	GAATCCTTTAGCAGTGGGTC C	TTTGTCTGCCTCTGATGTGG
CRTC2	hsa	GACAAGCTCTGACTCTGCCC	CCATCCAGAATACCCCCAC
CRTC3	hsa	AGATGTGGGTTTTGACCAGC	TGTTGAGGTCTTTGAACAGGC
SEC14l3	hsa	CAAGGGGTTGCTCTTCTCAG	TCCCTAGCCTCTCTGTCTGC
MUC5AC	hsa	GCCTTCACTGTACTGGCTGA G	AGGGTCTGAAGATGGTGACG
FOXJ1	hsa	TCGTATGCCACGCTCATCTG	CTTGTAGATGGCCGACAGGG
HPRT	hsa	TTGTTGTAGGATATGCCCTTG A	TCTCATCTTAGGCTTTGTATTTTG C
CREB1	mmu/hsa	CCCAGCAACCAAGTTGTTGTT	CTGCCTCCCTGTTCTTCATTAGAC
Crtc1	mmu	CGGGCTCCACACTCAACTA	TGCTCAGTTCCTTAGAGAGGCT
Crtc2	mmu	GTACAACGAGATGCCCGC	CTAAACAACTGCCCCTTCTCAG
Crtc3	mmu	GCATGAGTGTGGGGAACAG	TGGATGGAAGGGTTACTTCG
Sec14l3	mmu	CATGTGTCAGCGAAATCCC	TCTATGTCAATGCAGCGAGTG
Hprt	mmu	CAGGCCAGACTTTGTTGGAT	ACGTGATTCAAATCCCTGAAGT

Table S3: Primer sequences for qRT-PCR : *hsa = homo sapiens , mmu = mus musculus

SUPPLEMENTARY FIGURES & LEGENDS

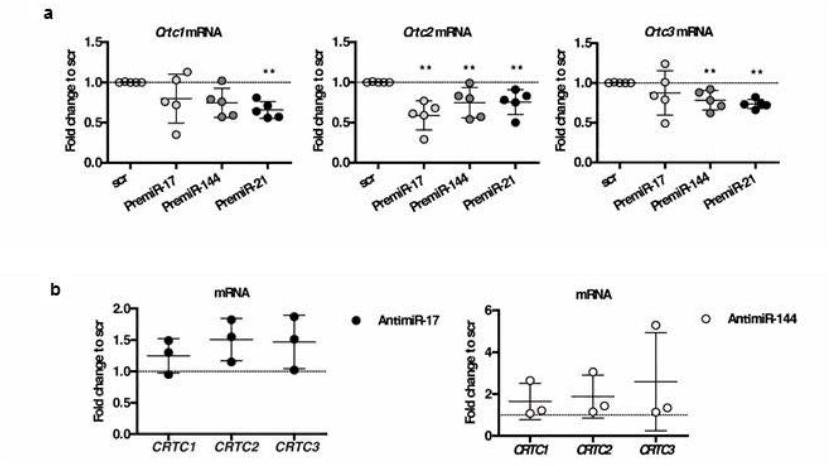

Fig. S1. miRNA-mediated regulation of *CRTCs* in vitro.

(a) qRT-PCR for *Creb1* & *Crtc1* and *Crtc3* after PremiR transfection of MLE-12 cells. (**b**), Transfection of 16-HBE14o^{-} cells with antimiRs. qRT-PCR for *Crtc1* and *Crtc3*, representative blot for n=3 independent experiments. All mean ± SD, Fold change to scrambled control (scr). Unpaired t-test *p<0.05; **p<0.01 vs. scrambled control.

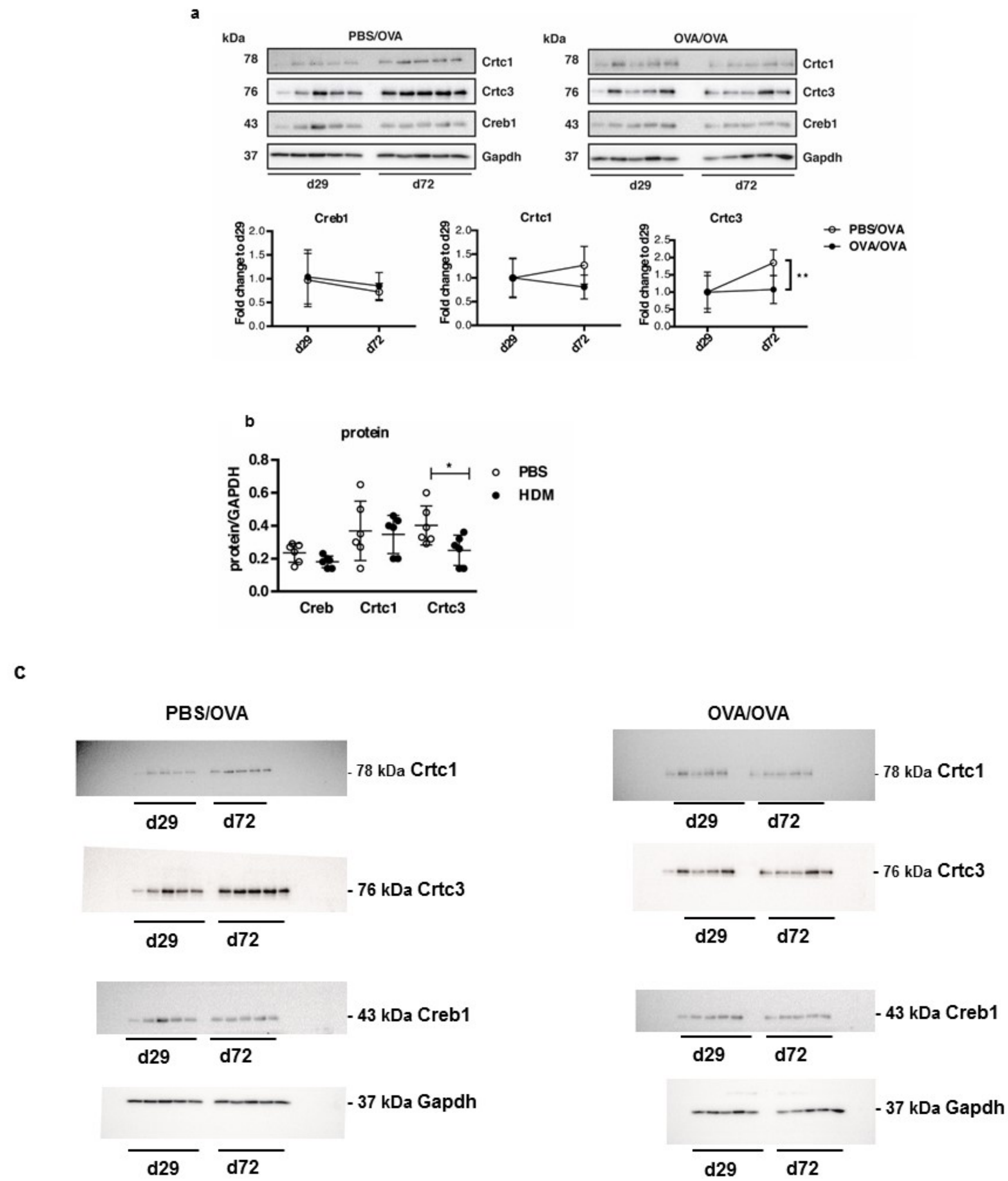

Fig. S2. Western Blot analysis of Creb1 and Crtc1-3 levels with respective densitometrical analyses in lung homogenate in **(a)** PBS/OVA or OVA/OVA treated animals on d72 vs d29, (n=5 mice per group) mean ± SD, fold change to d29. Blots have been cropped to improve clarity, see (c) for full-length images **(b)** HDM-treated mice vs. PBS treated controls, (n=6 mice per group). Volume intensity of proteins normalized to GAPDH. All mean ± SD, Mann-Whitney U *p<0.05; **p<0.01 vs. respective controls. **(c)** full-length images of western blots.

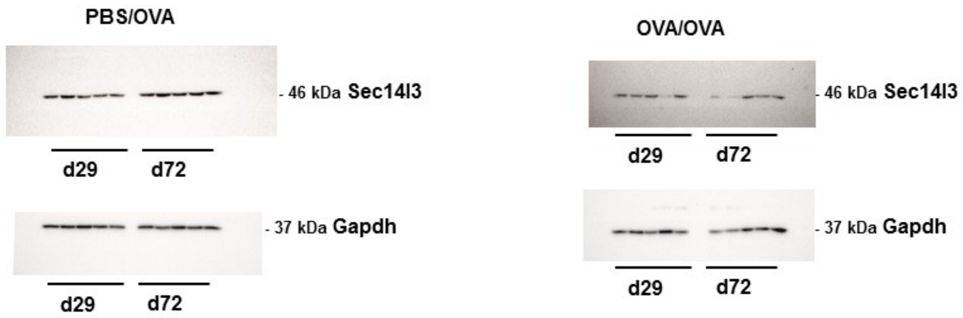

Fig. S3. Full length western Blots for Sec1l43 of lung homogenate upon OVA-induced allergic airway inflammation (shown in Fig. 5). PBS/OVA or OVA/OVA treated animals on d29 vs d72, (n=5 mice per group).

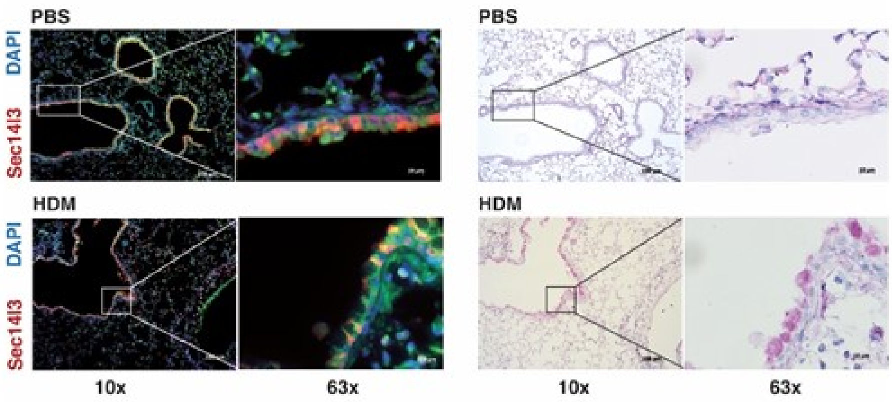

Fig. S4. Sec14l3^{+} cells are lost in HDM-induced allergic airway inflammation. Representative lung sections of HDM-treated mice (lower panel) vs. PBS-treated controls (upper panel): left panel shows Sec14l3 (red) vs. DAPI (blue), right panel shows PAS staining of the identical sections. Representative sections of 4 mice/group.

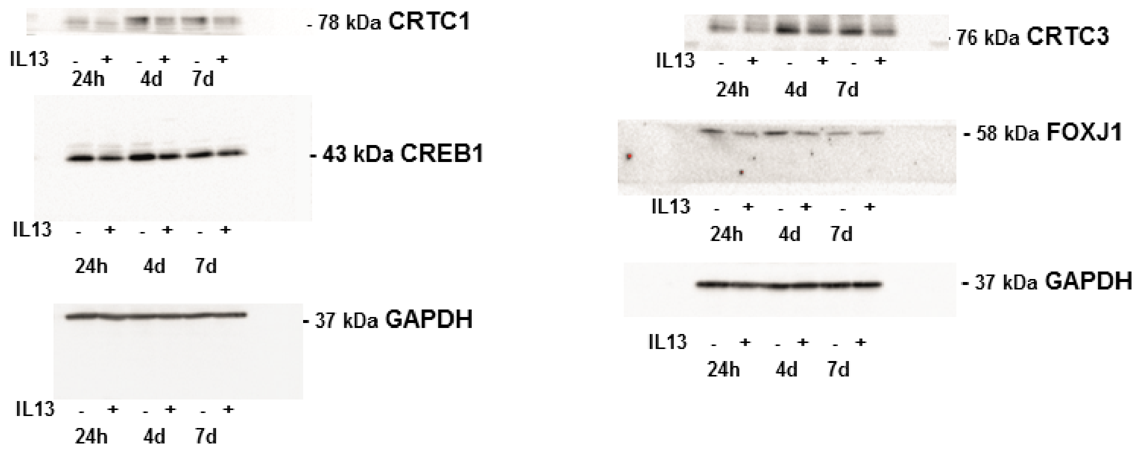

Fig. S5. Full length Western Blots for CREB1 & CRTC1-3 in primary NHBE cells (shown in Fig. 7). NHBE cells were treated with (+) or without (-) 10 ng/ml IL13 and analyzed after 24h, 4d or 7d.

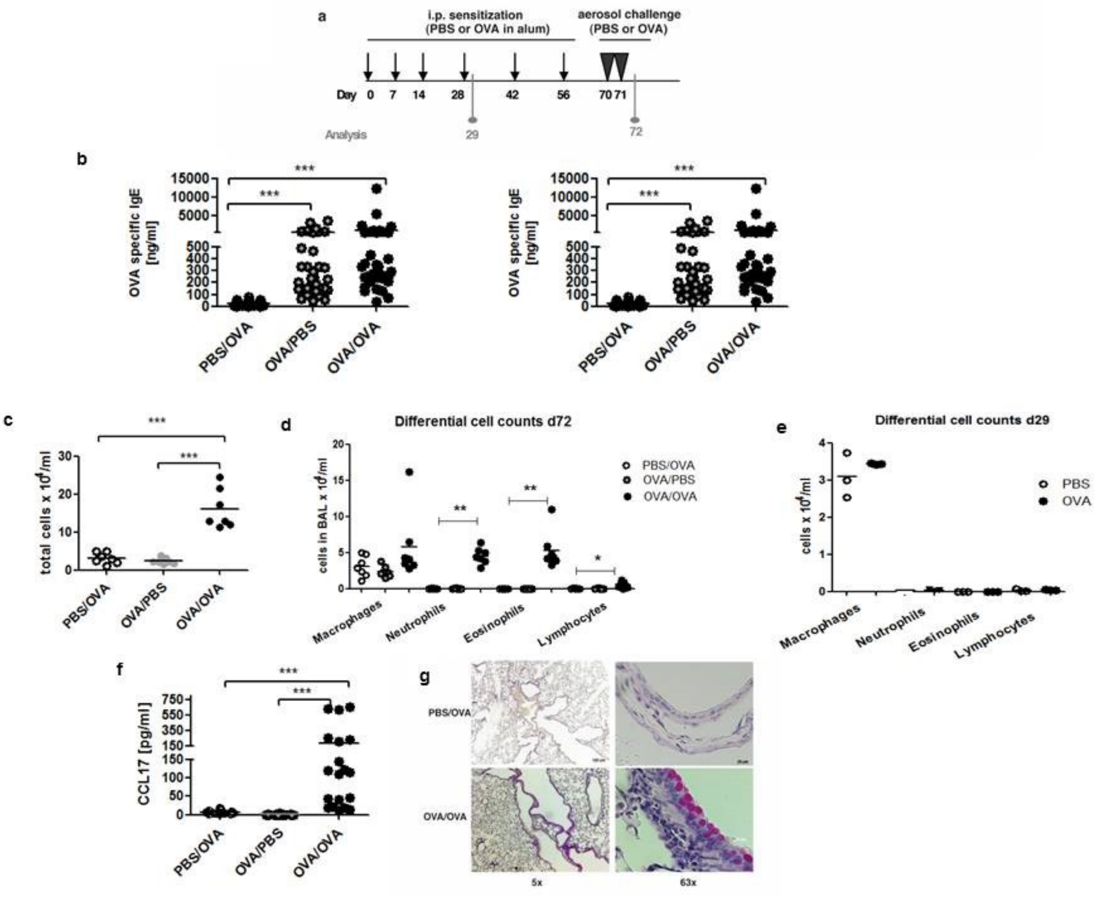

Fig. S6. Ovalbumin (OVA)-induced allergic airway inflammation

(a) Treatment scheme. **(b)** OVA-specific IgE (left) and IgG1 (right) in serum of PBS/OVA, OVA/PBS and OVA/OVA treated mice at d72. **(c)** Total and **(d)** Differential cell counts in BAL at d72. n=7 per group. **(e)** Differential cell count in BAL at d29. n=3 per group **(f)** CCL17 chemokine levels in BAL fluid on d72. **(g)** PAS stain of PBS/OVA and OVA/OVA. $Mucin^+$ goblet cells are depicted in pink. n = 35 per group. mean ± SD, Mann-Whitney U, ***p<0.001 vs. PBS/OVA.

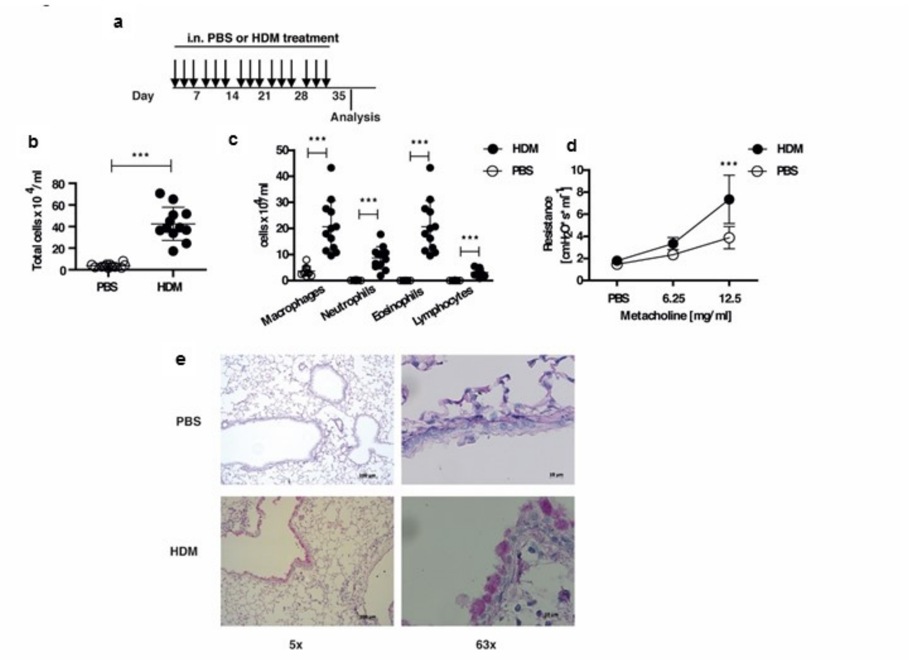

Fig. S7. House dust mite induced allergic airway inflammation.

(a) Treatment scheme for HDM vs. PBS-treated mice. **(b)** Total and (**c**) Differential cell counts in BAL. **(d)** Airway resistance after methacholine challenge, n=12/group, analysed by ANOVA+ Bonferroni. **(e)** PAS staining of PBS vs. HDM-treated mice. Mucin$^+$ goblet cells are depicted in pink. mean ± SD, Mann-Whitney U, ***p<0.001 vs PBS.
